# The Effect of Ultrasonic Agitation on the Seedless Growth of Cu on Ru-W Thin Films

**DOI:** 10.3390/ma16010167

**Published:** 2022-12-24

**Authors:** Rúben F. Santos, Bruno M. C. Oliveira, Paulo J. Ferreira, Manuel F. Vieira

**Affiliations:** 1 Department of Metallurgical and Materials Engineering, University of Porto, 4200-465 Porto, Portugal; 2 LAETA/INEGI—Institute of Science and Innovation in Mechanical and Industrial Engineering, 4200-465 Porto, Portugal; 3 International Iberian Nanotechnology Laboratory, 4715-330 Braga, Portugal; 4 Materials Science and Engineering Program, University of Texas at Austin, Austin, TX 78712, USA; 5 Mechanical Engineering Department and IDMEC, IST, University of Lisbon, 1749-016 Lisbon, Portugal

**Keywords:** Cu, Ru, W, diffusion barrier, seedless, electroplating, acidic, ultrasound, agitation

## Abstract

Ru attracted considerable attention as a candidate to replace TaN as a diffusion barrier layer for Cu interconnect metallisation. The addition of W improves the diffusion barrier properties of Ru but appears to weaken the adhesion strength between the barrier and Cu and the direct (seedless) electroplatability behaviour. Although Cu can be directly electroplated on near equimolar Ru-W thin films, no complete substrate coverage is obtained. The understanding of Cu electrocrystallisation on Ru–W is essential to develop methods of fabricating thin, continuous, and well adherent films for advanced interconnect metallisation, where Ru–W thin films could be used as diffusion barriers. This work studies the effect of ultrasonic agitation on the growth of Cu films electroplated on Ru–W, namely on the impact on substrate coverage. Film structure, morphology and chemical composition were evaluated by digital and scanning and transmission electron microscopies, and X-ray diffraction. The results show that Cu particles decrease with increasing current density, but when no electrolyte agitation is applied, substrate coverage is incomplete in the central region, with openings around larger Cu particles, regardless of current density. Under ultrasonic agitation, substrate coverage is remarkably improved. An active particle detachment mechanism is proposed as responsible for attaining improved substrate coverage, only possible at intermediate current density. Lower current densities promote growth over nucleation, whereas higher currents result in extensive hydrogen reduction/formation. Ultrasonic agitation also enhances a preferential Cu growth along <111> direction.

## 1. Introduction

Powerful and efficient integrated circuits (IC) such as central processing units (CPU) and graphic processing units (GPU) are possible due to a continuous miniaturisation of transistors, down to the nanoscale. Such evolution implies a continuous miniaturisation of all the structures inside an IC, including the interconnects. This translates into new challenges related to the materials being used for interconnect lining and metallisation, among others [1].

Interconnect metallisation with Cu has been the industry standard for more than 20 years now, since its introduction by IBM in the late 1990s. Cu suitably replaced Al interconnects because of its lower resistivity (ρ_Al_ = 2.65 × 10^−8^ Ω·m; ρ_Cu_ = 1.68 × 10^−8^ Ω·m) and higher electromigration resistance. The fabrication of Cu interconnects, however, requires different fabrication processes, different equipment, and the use of a lining to prevent Cu diffusion into the surrounding dielectric materials. TaN has been the industry standard material for that lining purpose, combined with a Ta adhesion layer sitting between TaN and Cu [2]. Although effective as a diffusion barrier, TaN has a relatively high resistivity (ρ_TaN_ ≈ 80 × 10^−8^ Ω·m [3]) that, associated with ever narrower interconnect vias and lines, increases overall interconnect resistivity, damping the electronic performance and eventually leading to interconnect disruption. Additionally, due to the terminal effect [4], Ta/TaN lining bilayers are not suitable for direct electroplating—the conventional method for Cu interconnect fill-up—requiring a thin Cu seed layer to be first deposited by physical vapour deposition (PVD) techniques. This seed layer is becoming more challenging to fabricate as dimensions shrink. To overcome these setbacks, fully metallic systems have been considered as alternatives to replace TaN as diffusion barrier layers.

Ru attracted considerable attention as candidate to replace TaN. Ru has a high melting point (T_m,Ru_ = 2607 K), displays more than a 10-fold lower resistivity (ρ_Ru_ ≈ 7.1 × 10^−8^ Ω·m) than TaN [5], has better adhesion to Cu [6,7], and is suitable for seedless Cu electroplating. Ru is also chemically inert and stable in conventional acidic electroplating solutions, contrary to other candidates such as Co-based systems, which are prone to dissolution in acidic media [8,9]. As for the task of preventing Cu diffusion, Ru performance can be competitive when coupled with other elements such as Ru–Co [10], Ru–Cr [11], Ru–Mn [12,13,14], Ru–Ta [12,15], and Ru–W [12,16]. Wojcik et al. [12] studied the performance of Ru, and Ru–W, Ta, Mn) thin films, finding that Ru–W (50:50) ranks among the best performing systems as a Cu diffusion barrier, up to 600 °C, whereas Ru alone fails at temperatures as low as 350 °C. A similar result was obtained by Yeh et al. [16] for Ru and near equimolar Ru–W thin films, with failure temperatures of 650 and 500 °C, respectively. The addition of W improves the diffusion barrier properties of Ru but appears to weaken the adhesion strength between the barrier and Cu and the direct (seedless) electroplating behaviour. Although Cu can be directly electroplated on near equimolar Ru–W thin films, no complete substrate coverage is obtained [17,18]. A recent work shows that the growth of large Cu particles during electrocrystallisation hinders nucleation on their vicinity, leaving substrate coverage gaps [18]. The adoption of Ru–W barrier layers as alternatives to TaN depends on their effectiveness for seedless interconnect metallisation. Since these layers can be produced by industry known processes such as PVD, their adoption would not be technologically disruptive but would open the door to better performing devices. Thus, the objective of this study is to enhance the substrate coverage through mechanical stimulus during Cu electrodeposition. While Ru–W seems an interesting candidate system as a diffusion barrier layer, the direct Cu electrodeposition behaviour and the characteristics of electrodeposited Cu films on Ru–W lack a fundamental understanding. To the best of our knowledge, the effect of ultrasonic agitation on the electrodeposition behaviour of Cu on Ru–W thin films has never been reported. For this reason, the present work aims at improving the seedless Cu electroplating on Ru–W using ultrasonic electrolyte agitation.

## 2. Experimental

### 2.1. Substrate Preparation

A ≈ 100 nm thick SiO_2_ layer was grown on the surface of a p-type B-doped Si (100) wafer (Silicon Valley Microelectronics, Santa Clara, CA, USA) by plasma enhanced chemical vapour deposition (PECVD) with high radio frequency in a CVD MPX chamber (SPTS Technologies Ltd., Newport, UK). Afterwards, the wafer was cut in smaller portions (15 mm × 15 mm) whereon Ru–W films were co-deposited by DC magnetron sputtering in an ultra-high vacuum sputtering system (Kenosistec, Binasco, Italy). Power biases of 40 and 30 W were applied to Ru and W targets (99.95% purity, Testbourne Ltd., Basingstoke, UK), respectively. Deposition was performed in a chamber at 6.9 × 10^−1^ Pa with Ar influx of 20 sccm for 600 s. Sputtering parameters were selected in order to produce a ≈20 nm Ru–W film with near-equimolar composition, based on a previous work [18].

### 2.2. Cu Electroplating

**Figure 1:**
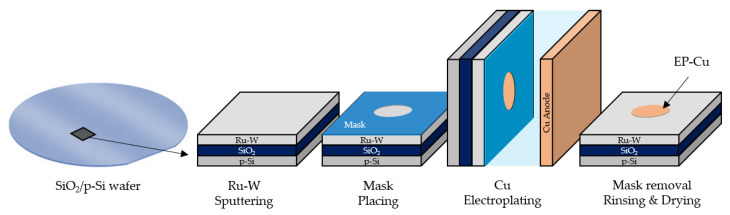
Schematic sequence of substrate preparation and Cu electroplating.

Cu electroplating was conducted in an acidic electrolyte of 0.05 M CuSO_4_∙5H_2_O (99.995%, Sigma-Aldrich, St. Louis, MO, USA), 0.05 M H_2_SO_4_ (Honeywell/Fluka, Charlotte, NC, USA), 1 mM NaCl (Honeywell/Fluka, Charlotte, NC, USA) and 300 ppm poly(ethylene glycol) 600 (Fluka Chemie GmbH, Buchs, Switzerland) prepared with deionized water. A two-electrode plating cell was assembled, with the Ru–W/SiO_2_/Si substrates as the cathode and a 3 mm-thick copper plate as the anode, which was positioned parallel to the cathode at a distance of 65 mm. Electrodeposition took place using a Gamry potentiostat/galvanostat Interface 1000E (Gamry Instruments, Warminster, PA, USA) at room temperature with and without ultrasonic (35 kHz) agitation in a Sonorex Super (Bandelin Electronic GmbH & Co., Berlin, Germany). Prior to electrodeposition, a polyvinyl chloride mask was applied on the substrates limiting the exposed area to a circle of 0.50 cm^2^. After electroplating, the substrates were gently rinsed in deionised water and dried with a soft Ar blow. A descriptive illustration of substrate preparation and electroplating is shown in Figure 1.

### 2.3. Structural and Chemical Characterisation of Substrate and Cu Films

The surfaces of the as-sputtered Ru–W/SiO_2_/Si substrates were observed by scanning electron microscopy (SEM) (Thermo Fisher Scientific Quanta 400FEG ESEM, Thermo Fisher Scientific, Hillsboro, OR, USA), and their chemical composition estimated by energy-dispersive X-ray spectroscopy (EDS) (EDAX Genesis X4M, AMETEK, Berwyn, PA, USA). Ru–W film structure was assessed by grazing incidence X-ray diffraction (GIXRD) at an angle of 1.5°, using Cu Kα radiation (λ = 1.54187 Å), and a step of 0.04 °·s^−1^ (Bruker D8 Discover, Bruker Corporation, Billerica, MA, USA), whereas the Cu films were analysed with Bragg–Brentano configuration with a step of 0.005° (X’Pert Pro MRD, Malvern Panalytical, Worcestershire, UK). The Cu films were also observed by digital microscopy (Leica DVM6, Leica Microsystems GmbH, Wetzlar, Germany) and further analysed by SEM (Thermo Fisher Scientific Quanta 650FEG ESEM, Thermo Fisher Scientific, Hillsboro, OR, USA). A cross-sectional lamella was prepared by focused ion beam, FIB (Thermo Fisher Scientific Helios 450S) and observed by scanning transmission electron microscopy, STEM (Thermo Fisher Scientific Titan G2 ChemiSTEM). Surface roughness of Ru–W films was measured by atomic force microscopy, AFM (Veeco Metrology Multimode, Veeco Instruments Inc., Oyster Bay, NY, USA), with a Bruker TESPA-V2 tip, and with the *NanoScope* software 6.13R1 (Veeco Instruments Inc.). Image analyses were performed on *ImageJ* software version 1.51p (National Institutes of Health, Bethesda, MD, USA).

## 3. Results and Discussion

**Figure 2:**
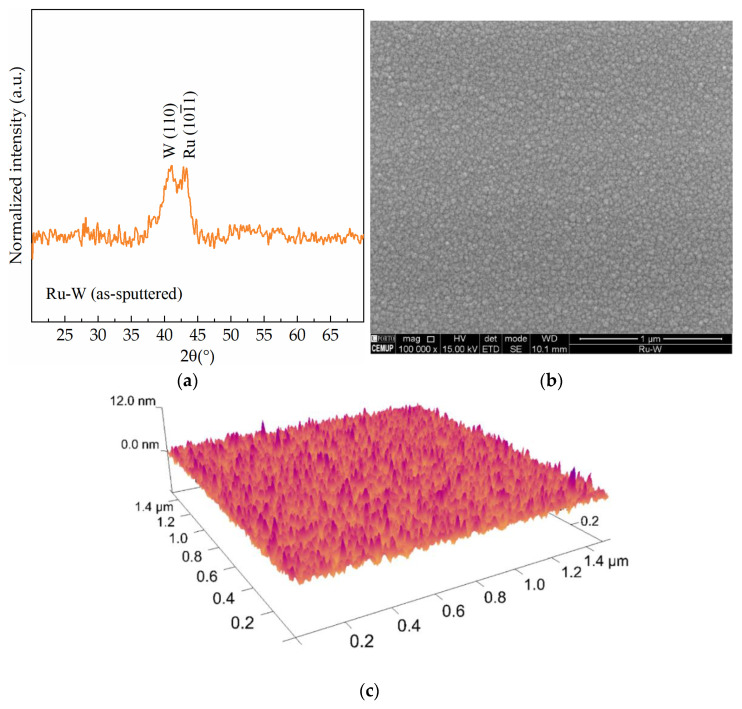
As-sputtered Ru–W film X-ray diffractogram (**a**), and its surface observed by SEM (**b**) and by AFM (**c**).

The conditions to fabricate near equimolar Ru–W thin films were determined in a previous work [18] and herein replicated. In terms of structure, the Ru–W appears to have reduced crystallinity given by the short, broad XRD peaks (Figure 2a). In fact, the addition of W to Ru decreases the crystallinity in the film, an effect observed in other systems such as Ru–Cr [15]. The surface of the as-sputtered Ru–W thin films is relatively smooth (Figure 2b), with an average roughness, $S_{a}$, of 0.67 nm, measured by AFM over a 1.5 × 1.5 μm (Figure 2c). Electroplating was performed using direct current (DC) with densities, J, between 5 and 15 mA·cm^−2^, for 300 and 900 mC of transferred charge, Q. All current density and transferred charge combinations were tested with and without ultrasonic agitation (UA), summarised in Table 1.

**Figure 3:**
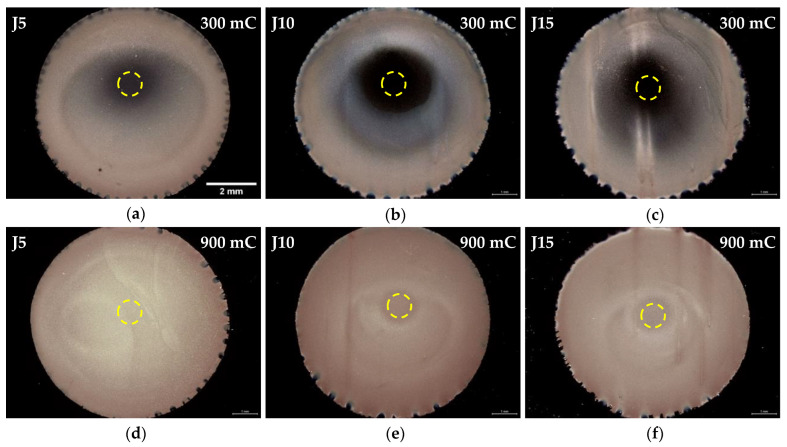
Digital microscopy images of the as-deposited Cu films at 5 (**a**), 10 (**b**), and 15 mA·cm^−2^ (**c**) for 300 mC and the same current densities (**d**), (**e**), and (**f**), respectively, for 900 mC, without electrolyte agitation.

**Figure 4:**
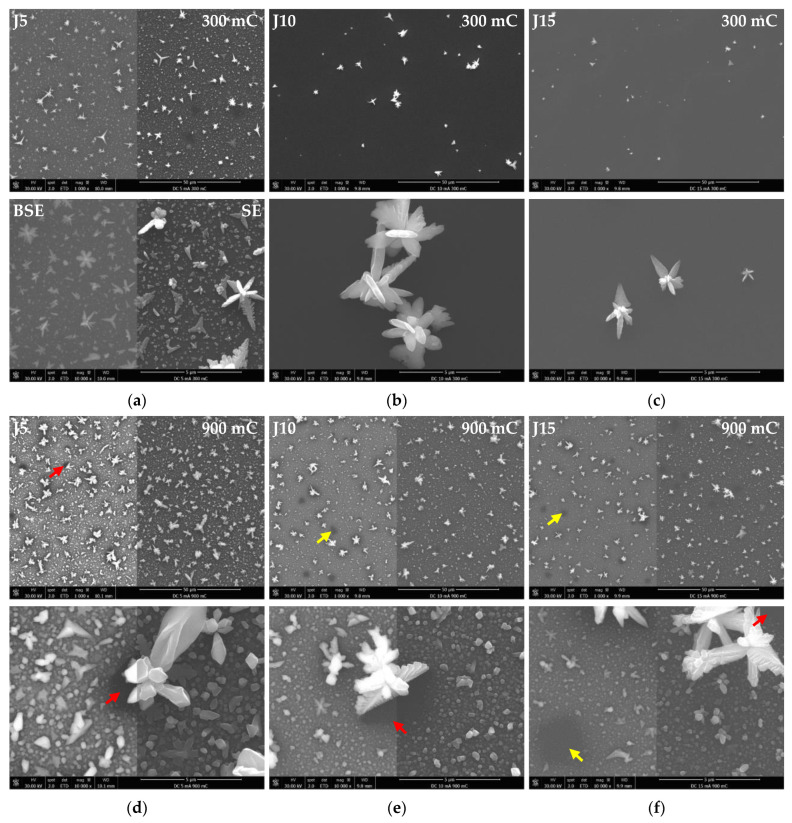
SEM images of the central regions of the as-deposited Cu films with current densities of 5 (**a**), 10 (**b**), 15 mA·cm^−2^ (**c**) for 300 mC and at the same current densities in (**d**), (**e**), and (**f**), respectively, for 900 mC, without electrolyte agitation. BSE images are overlaid on SE images in some of the figures.

As-deposited Cu/Ru–W surface images are displayed in Figure 3 for both 300 and 900 mC of transferred charge without electrolyte agitation. Considering substrate coverage, the plating process starts on the outer limits of the substrate and progresses inwards as the time unfollows. The central regions (yellow circles) of the substrate remain mostly uncovered for Q = 300 mC (Figure 3a–c), whereas at 900 mC (Figure 3d–f) macroscopic coverage is achieved. Small hydrogen bubbles form and grow close to the interface with the mask, due to H^+^ co-reduction. Their appearance is noticeable during the electrodeposition process, leaving bite-shaped marks around the Cu film that appear to increase in size with current density, attributed to a more intense hydrogen formation. This process increases with J, consuming a non-negligible part of the transferred charge that is not used for Cu electrocrystallisation, explaining the comparatively better substrate coverage obtained at 5 mA·cm^−2^ (Figure 4a–c). However, even in J5 the substrate remains considerably uncovered when observed by SEM (Figure 4a); in here, early growth Cu particles are observed isolated from each other across the bare Ru–W film. This is more clearly seen by the backscattered electron (BSE) SEM images overlaid on secondary electrons (SE) SEM images. It is worth mentioning that in ideal electroplating conditions, i.e., infinite number of active sites on the substrate for nucleation followed by uniform growth and absence of hydrogen ion co-reduction (Faradaic efficiency = 1), a Q value of 300 mC would suffice to form a ≈ 110 nm film across the substrate.

**Figure 5:**
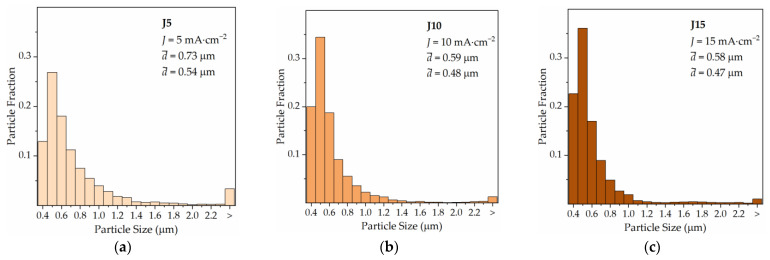
Particle size distribution for current densities of 5 (**a**), 10 (**b**), and 15 mA·cm^−2^ (**c**) at 900 mC, without electrolyte agitation. Only particles larger than 370 nm were measured and considered for descriptive statistics analysis.

With a 3-fold increase in electroplating time (Q = 900B mC), far better coverage is achieved in the central region of the substrate, with Cu particles of different sizes densely grown on the surface, except in the vicinity areas of large particles (red arrows in Figure 4d–f). Given their size, associated with a small contact area on the substrate, large Cu particles can easily detach and fall off the surface when subjected to the mechanical perturbation, such as during rinsing, drying, and transporting of the specimens, and even during electrodeposition. However, their nucleation and growth sites can still be identified by the coverage gaps left behind (yellow arrows in Figure 4e,f). Such incomplete substrate coverage scenario was observed in a previous study, and a mechanism for its occurrence was proposed [18]. The abnormal growth of some Cu particles presumably responsible for incomplete substrate coverage is not eliminated by increasing the current density. In spite of this, higher values of J favour nucleation over growth, effectively reducing the particle size, given by the smaller average ($\bar{d}$) and median ($\tilde{d}$) particle size values (Figure 5).

**Figure 6:**
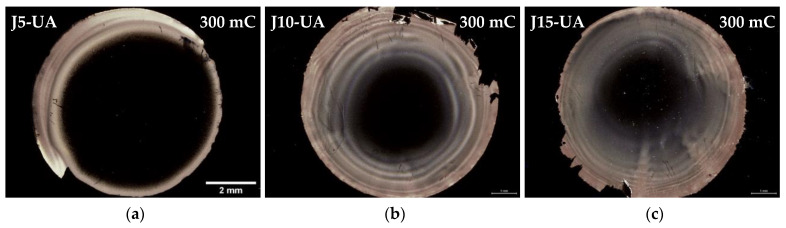

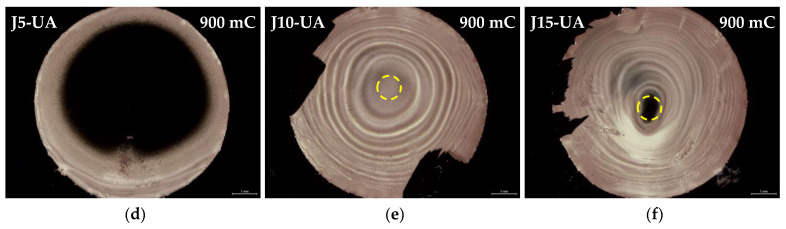
Digital microscopy images of the as deposited Cu films at 5 (**a**), 10 (**b**), and 15 mA·cm^−2^ (**c**) for 300 mC and the same current densities (**d**), (**e**), and (**f**), respectively, for 900 mC, under ultrasonic electrolyte agitation.

**Figure 7:**
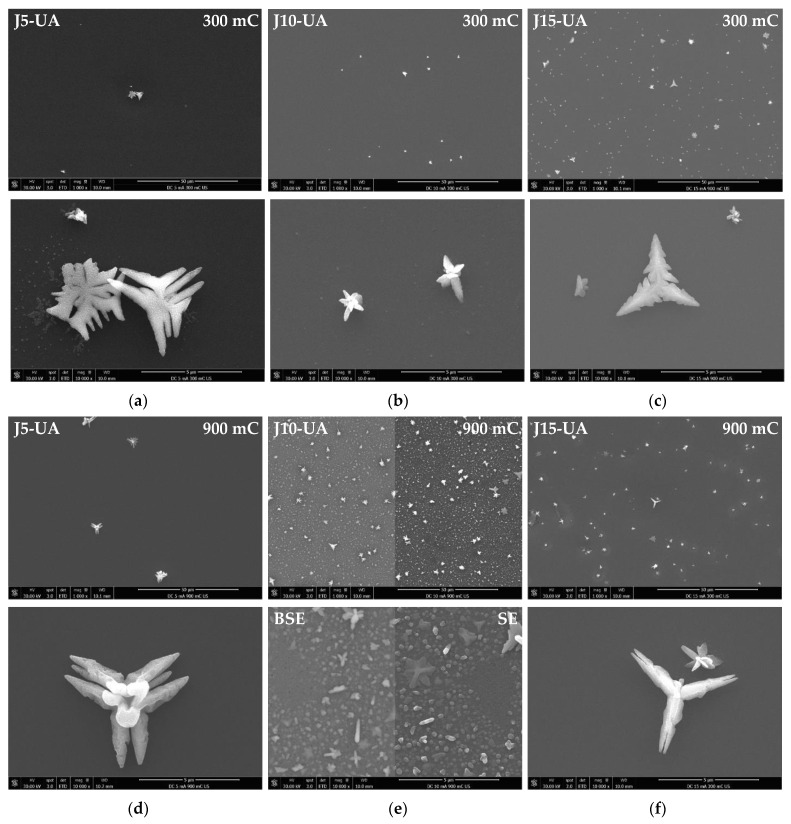
SEM images of the central regions of the as deposited Cu films with current densities of 5 (**a**), 10 (**b**), 15 mA·cm^−2^ (**c**) for 300 mC and at the same current densities in (**d**), (**e**), and (**f**), respectively, for 900 mC, under ultrasonic electrolyte agitation.

Macroscopic substrate coverage decreases when ultrasonic agitation is applied during the electroplating process (Figure 6a–c). It is reasonable to assume that the mechanical waves generated by UA constitute a strong stimulus for the detachment of many Cu particles, especially the larger/more massive ones. Thus, under such conditions, the amount of Cu mass that remains attached to the substrate after plating is expected to be lower than that obtained when no electrolyte agitation is applied, for identical values of J and Q. It is interesting to note that under UA, substrate coverage increases with current density, contrary to what happens when agitation is absent; without agitation, the macroscopical substrate coverage is mainly limited by the extent of hydrogen co-reduction, whereas when UA is applied the extent of Cu particle detachment, due to mechanical vibration, plays a dominant role when lower current densities are used. Since lower values of J favour growth over nucleation resulting in larger particles, they should result in a larger loss of mass when UA is applied. This outcome is clearly revealed by comparison of Figure 2a with Figure 6a and explains the poor substrate coverage obtained in J5-UA. For higher values of J, not only particles are smaller and comparatively less prone to detachment under UA, but also hydrogen co-reduction plays a strong role, resulting in substrate coverage differences (J15 in Figure 2c vs. J15-UA in Figure 6c) that are far less expressive. The agitation of the electrolyte with ultrasonic waves promotes a faster replenishing of Cu^2+^ ions to the substrate–electrolyte interface, decreasing the extension of hydrogen co-reduction. The uncovered central region of the substrates at 300 mC under UA is also revealed by SEM, with a few Cu particles scattered across the surface, being the particle density higher with higher values of J (Figure 7a–c).

**Figure 8:**
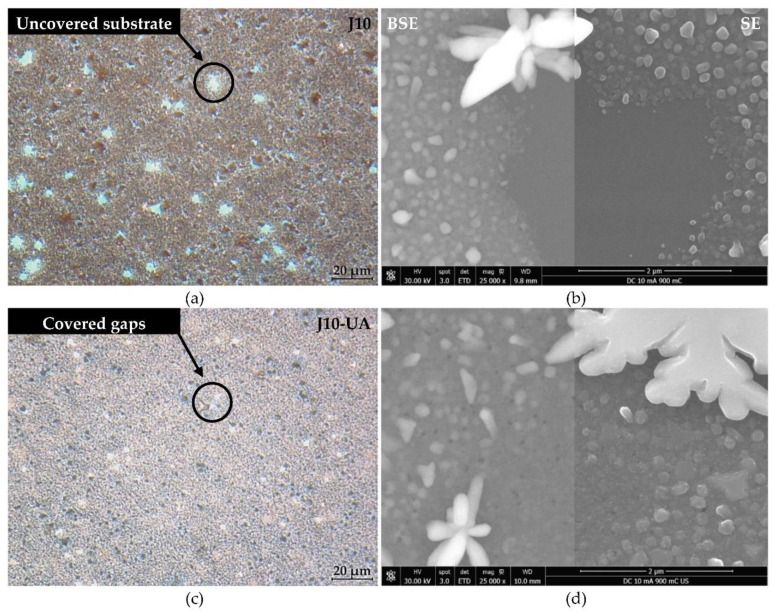
Digital optical microscopy images of the as-deposited Cu film obtained at 10 mA·cm^−2^ without agitation (**a**) and under ultrasonic electrolyte agitation (**c**). The same films observed at higher magnification by SEM in (**b**) and (**d**), respectively.

**Figure 9:**
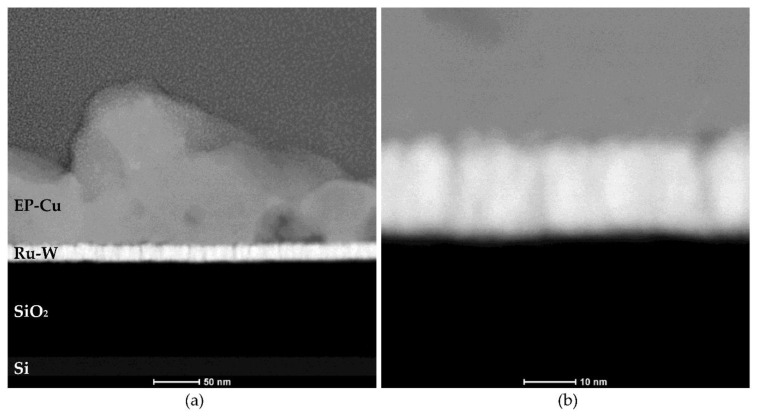
HAADF image of the Cu/Ru–W/SiO_2_/Si stack cross-section (**a**) and higher magnification of the same zone showing interfacial continuity between electroplated Cu film and Ru–W substrate (**b**).

**Figure 10:**
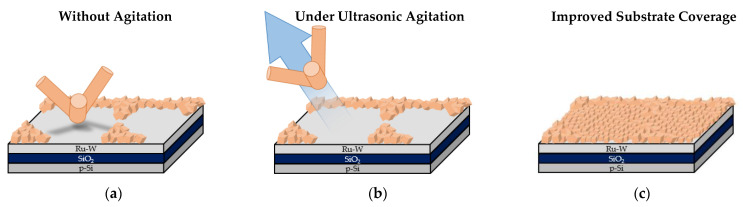
Electrocrystallisation model for Cu on Ru–W thin films with and without ultrasonic agitation. Incomplete substrate coverage around large Cu particles without ultrasonic agitation (**a**), Cu particle detachment via mechanical stimulus (**b**), and filling of the coverage gaps after Cu particle removal (**c**).

A 3-fold increase in the transferred charge (Q = 900 mC) for J5-UA fails to produce anything close to a complete substrate coverage, given by a rather unimpressive improvement over its 300 mC counterpart (Figure 6d). The extra charge supplied to the substrate is likely offset by particle detachment under UA. The additional transferred charge is consumed to promote the growth of the Cu film on the outer regions of the substrate. A major improvement is observed for J10-UA where both particle detachment and hydrogen co-reduction are not too intense, and an improved substrate coverage is achieved (Figure 6e). Under UA, any hydrogen bubbles are promptly dissipated from the substrate’s surface, not being allowed to grow, and imprint bite-shape marks around the Cu films; such was the case when agitation is not used. Nevertheless, it is plausible that at higher current density (J = 15 mA·cm^−2^) hydrogen reduction becomes strong enough to prevent complete coverage, as is seen for J15-UA (Figure 6f). This scenario is confirmed microscopically by SEM, with the central substrate regions of the J5-UA (Figure 7d) and J15-UA (Figure 7f) specimens fairly exposed/uncovered, whereas J10-UA displays an improved substrate coverage (Figure 7e). It is worth mentioning that despite the central regions of the substrate in J5-UA and J15-UA both being uncovered, the latter specimen has a much better coverage overall. This fact suggests that Cu particle detachment (more likely to occur in J5-UA) exerts a more pronounced contribution to the loss of transferred charge than hydrogen co-reduction does (more likely to take place in J15-UA). A closer look at the Cu film surface for J10-UA shows no substrate coverage gaps, such as those found around large particles when no agitation is used (Figure 4e). Under UA, the firstly formed large Cu particles are detached/removed from the substrate, leaving an exposed area, whereon many Cu nuclei readily form and grow. The process is repeated across the surface until all the substrate openings are filled. Eventually, additional large Cu particles grow but this time on top of a pre-existing thin Cu film. This is clearly observed in digital microscopy where the uncovered portions of the Ru–W substrate (gaps) are revealed by bright bluish zones (Figure 8a) and in higher magnification SEM images (Figure 8b). When UA is applied, the gaps are filled with Cu film, shown as bright whitish zones (Figure 8c). It is noteworthy that these Cu-filled gaps display a substantially finer/smoother surface (Figure 8d), hence appearing brighter in digital microscopy. High-angle annular dark-field (HAADF) imaging obtained in STEM for the J10 (900 mC) cross-section reveal that aside from the coverage gaps, Cu film displays good interfacial continuity with the Ru–W substrate with thickness varying between tens to several hundred nanometres (Figure 9). The proposed mechanism for the effect of UA on substrate coverage is illustrated in Figure 10. A concerning aspect regarding the use of UA is the impact on Cu film adhesion, since the films produced under such condition were partially detached from the substrate during mask removal, particularly for current densities of 10 and 15 mA·cm^−2^.

**Figure 11:**
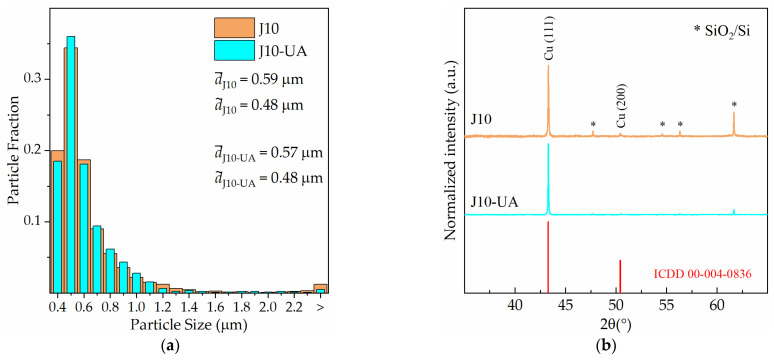
Particle size distributions for current density of 10 mA·cm^−2^ with (J10-UA) and without (J10) agitation (**a**), and their respective X-ray diffractograms (**b**).

The use of UA does not appear to have an impact on the average particle size (Figure 11a), but it is interesting to note what it does to the electrocrystallisation of Cu on Ru–W in terms of preferential Cu growth along <111> directions. This preferential orientation, which is typically present in non-epitaxial growth [19], minimises interfacial energy and is perceived by a lower (200)/(111) peak intensity ratio, $I_{\left(200\right)}$, when comparing the EP-Cu XRD ($I_{\left(200\right),J10}$ = 4.4%) with the ICDD Cu pattern (00-004-0836) ($I_{\left(200\right)}$ = 46.0%). When UA is applied, such preferential growth is enhanced ($I_{\left(200\right),\;J10-UA}$ = 0.8%), meaning an enhancement in the preferential <111> growth (Figure 11b). This is an interesting microstructural aspect for Cu interconnect due to the lower resistivity and higher electromigration resistance of the {111} planes [20].

## 4. Conclusions

Cu was directly electroplated on Ru–W thin films with near equimolar composition at different current densities and two values of transferred charge, with and without ultrasonic agitation. When no agitation is used, hydrogen co-reduction is a key factor limiting macroscopical substrate coverage, which is lower for higher current density. Microscopically, improved substrate coverage is hindered by abnormal Cu particle growth, regardless of current density. Higher current density reduces average Cu particle size but promotes a more intense hydrogen co-reduction. Under ultrasonic agitation, an active Cu particle detachment reduces macroscopic substrate coverage, especially when particles grow larger, at lower current density. Under an intermediate intensity of hydrogen co-reduction and particle detachment, established by applying ultrasonic agitation at intermediate current density, a continuous Cu film grows, with microscopical continuity. Ultrasonic agitation enhances the preferential growth of Cu film along <111> directions.

## Figures and Tables

**Table 1:** Specimen tag names and respective electroplating conditions.

Specimen Tag Names	J mA·cm^−2^	Q mC	Ultrasonic Agitation
J5	5	300 900	No
J10	10		
J15	15		
J5-UA	5		Yes
J10-UA	10		
J15-UA	15		

## Data Availability

The data presented in this study are available on request from the corresponding author.
